# Preoperative Arithmetic Hip-Knee-Ankle Angle as a Predictor of Postoperative Leg Alignment in Medial Oxford Unicompartmental Knee Arthroplasty

**DOI:** 10.7759/cureus.56046

**Published:** 2024-03-12

**Authors:** Takafumi Hiranaka, Yasuhiro Fukai, Sho Tanaka, Takahiro Okajima, Yuya Ishida, Motoki Koide, Takaaki Fujishiro, Koji Okamoto

**Affiliations:** 1 Department of Orthopaedic Surgery and Joint Surgery Centre, Takatsuki General Hospital, Takatsuki, JPN

**Keywords:** joint line, alignment, unicompartmental, arthroplasty, knee

## Abstract

Introduction

This study aimed to evaluate whether the arithmetic hip-knee-ankle angle (aHKA) can be used to predict the postoperative HKA.

Methods

This study included 248 knees in 166 patients who underwent Oxford unicompartmental knee arthroplasty (UKA) between February 2021 and November 2022. Through preoperative and postoperative long-leg radiography, the medial proximal tibial angle (MPTA) and the lateral distal femoral angle (LDFA) were expressed as the deviation from the perpendicular line to the mechanical axes, and the mechanical HKA (mHKA) was defined as the angle between the femoral and tibial mechanical axes. Using the MPTA and LDFA, the arithmetic HKA (aHKA; MPTA + LDFA) and the joint line obliquity (JLO; MPTA − LDFA) were calculated, and the preoperative and postoperative values were compared.

Results

The preoperative aHKA and the postoperative mHKA values were similar (−0.38° ± 2.96°) and significantly smaller than the difference between the preoperative and postoperative mHKAs (4.58° ± 3.60°, *P* < 0.05). Meanwhile, the MPTA tended to be varus, and the LDFA tended to be valgus. Eventually, the JLO inclined more medially from −6.33° ± 3.42° preoperatively to −8.97° ± 3.92° postoperatively, representing a significant difference (*P* < 0.05).

Conclusion

The preoperative aHKA was similar to the postoperative mHKA. Therefore, it can be regarded as a predictor of postoperative leg alignment after Oxford UKA. Meanwhile, there was a medial incline of the joint line. Further investigation is required to evaluate the effect of such a joint line alteration.

## Introduction

Medial compartment osteoarthritis (OA) of the knee is one of the most common diseases, for which isolated medial compartmental arthroplasty is an effective treatment [1]. In addition to a short recovery time, its other reported benefits include low invasion and mortality and retained kinematics, with satisfactory long-term survival and patient satisfaction [2-5]. Oxford medial unicompartmental knee arthroplasty (UKA) is a resurfacing surgery based on ligament balancing, which aims to restore the patient-specific constitutional alignment (CA) rather than to reconstruct the neutral alignment, such as mechanically aligned total knee arthroplasty (TKA) [6-8]. If a patient’s CA can be predicted preoperatively, it will be beneficial to evaluate whether the patient’s alignment is correctly restored.

In TKA, the personalized alignment (PA) approach is represented by the kinematic alignment (KA). TKA [9-11] and its deliveries, such as restricted KA [12,13] and functional alignment [14,15], have emerged as a research hotspot. In PA TKA, the postoperative leg alignment is intended to be CA [16]. With the increasing interest in PA, efforts have been devoted toward determining how to find an individual’s optimal alignment [9]. Recently, CAs have been expressed by the arithmetic hip-knee-ankle angle (aHKA), which is calculated as the medial proximal tibial angle (MPTA) minus the lateral distal femoral angle (LDFA) [16,17]. Meanwhile, the joint line obliquity (JLO), calculated as MPTA + LDFA, has been proposed as a means to express other characteristics of an individual’s knee [16]. In the field of UKA, Mullaji et al. [6] reported that the postoperative alignment is similar to that of the opposite healthy leg in patients with unilateral OA. However, most OA patients have bilateral OA. For such patients, aHKA can be a reliable predictor of their CA and postoperative alignment after Oxford medial UKA.

This retrospective study aimed to evaluate the relationship between the postoperative HKA and the preoperative aHKA. We hypothesized that the aHKA can be used to predict the postoperative alignment after Oxford medial UKA because it aims to restore the pre-arthritic alignment by comparing the preoperative aHKA and the postoperative HKA. If this hypothesis is proven, the postoperative leg alignment can be predicted, which can be considered similar to the CA.

## Materials and methods

This retrospective study was performed according to the Declaration of Helsinki and with the approval of the ethics committee of our institution (No. 2021-23), as well as the informed consent of every patient. We considered 304 knees in 211 consecutive patients who underwent Oxford UKA for medial arthritis of the knee in our hospital between October 2022 and March 2023. All the patients underwent routine radiography: anteroposterior weighted, varus and valgus stressed anteroposterior at 20° knee flexion, Rosenburg, lateral, skyline, and long-leg standing radiographs. The inclusion criteria of the study were: (i) a confirmed diagnosis of medial arthritis of the knee that fulfills the indication of Oxford medial UKA as detailed below and (ii) a unilateral or bilateral simultaneous operation.

The indication of Oxford UKA was full cartilage defect of the medial compartment confirmed in the preoperative anteroposterior weighted, Rosenburg, or varus stress radiographies, and intact cartilage of the lateral compartment confirmed in the preoperative anteroposterior weighted, Rosenburg, or valgus stress radiographies with a functioning anterior cruciate ligament, which indicates that the bone erosion does not locate posteriorly on the tibial articular surface through lateral radiography, and the patellofemoral arthritis is acceptable (no lateral subluxation, bone defect, or grooving) [18]. Decisions were made on the basis of a radiological decision aid [19]. If we were unsure of the suitability of UKA for a knee, a backup TKA was prepared and converted into it depending on the intraoperative finding.

The exclusion criteria for this study were combined surgery (such as simultaneous lateral UKA or patellofemoral replacement, also known as bi-compartmental arthroplasty), the use of a fixed bearing because the gap was not accurately balanced in the fixed-bearing cases, previous operations such as hip and knee arthroplasty or osteotomy, previous fractures, and inadequate images.

Standing long-leg radiography was performed two weeks postoperatively. Then, the following evaluation was performed on the included patients.

Operation procedures and postoperative management

The operation procedures were performed using a microplasty instrumentation set. The flexion and extension gaps were adjusted within 1 mm using a feeler gauge and an incremental mill [20]. All the operations were performed by the senior author or under his supervision. Supported gait and range-of-motion exercises were encouraged as tolerated.

Preoperative radiographical evaluation

Preoperatively, the LDFA and MPTA were measured, and the value was expressed as the deviation from the perpendicular line to the mechanical axis (varus: −, valgus: +). The mechanical HKA (mHKA) was defined as the angle between the femoral and tibial mechanical axes.

Postoperative radiographical evaluation

**Figure 1:**
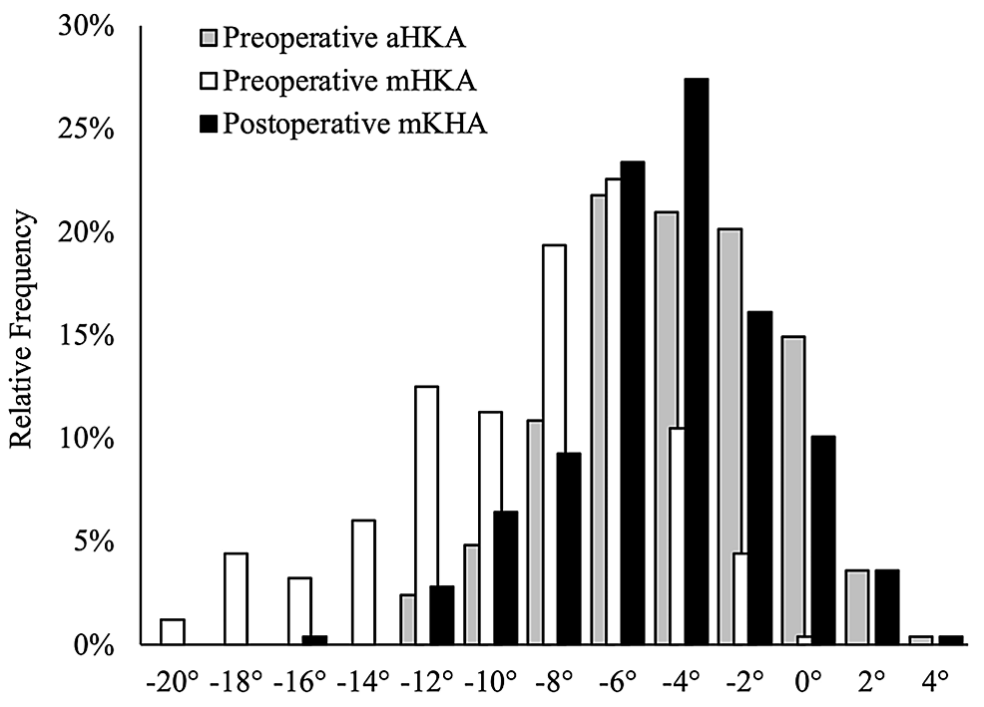
Distribution of the preoperative arithmetic hip-knee-ankle angle, the preoperative mechanical hip-knee-ankle angle and the postoperative mechanical hip-knee-ankle. The preoperative aHKA shows a similar distribution to the postoperative mHKA. On the other hand, the distribution of the preoperative mHKA tended to be more varus than the postoperative mHKA. aHKA: arithmetic hip-knee-ankle angle, mHKA: mechanical hip-knee-ankle.

Postoperatively, the joint line for LDFA and MPTA calculation was defined as the tangential line to the component surface that passes through the midpoint of the lateral compartment (Figure 1). In this evaluation, the thicknesses of the femoral and tibial lateral cartilage are considered to be similar. Regarding the medial condyle, the medial joint surface is reproduced, theoretically, because the UKA is a resurfacing surgery.

Calculation of the measurements

We also calculated the aHKA as the sum of the MPTA and the LDFA (varus: −; valgus: +) and the JLO as the MPTA minus the LDFA (apex-distal or medially inclined: −; apex-proximal or laterally inclined: +) (Figure 1). To compare the predictability of the mHKA and the aHKA preoperatively, their differences from the postoperative mHKA were calculated (mHKA-dif and aHKA-dif, respectively).

Statistical analysis

The data are expressed as the mean ± SD of all the measurements. The paired t-test was conducted to compare two measurements. The Pearson correction coefficients were used to evaluate the relationship between the preoperative aHKA and the postoperative mHKA and between the preoperative mHKA and the postoperative mHKA. The measurements of the LDFA, MPTA, and mHKA showed excellent intra- and inter-observer repeatability, and all the intra-class correlation coefficients were greater than 0.90. All the statistical analyses were performed using the Easy R application running on R (RStudio, Boston, MA) [21], except for the power analysis, which was carried out using G*Power (Heinrich Heine University Düsseldorf, Düsseldorf, Germany). A power of 0.8 was expected on the basis of the pre-specified significance level of α < 0.05, assuming a medium effect size. The estimated sample size was four knees, indicating that the sample size was significant. P-values less than 0.05 were considered statistically significant.

## Results

**Figure 2:**
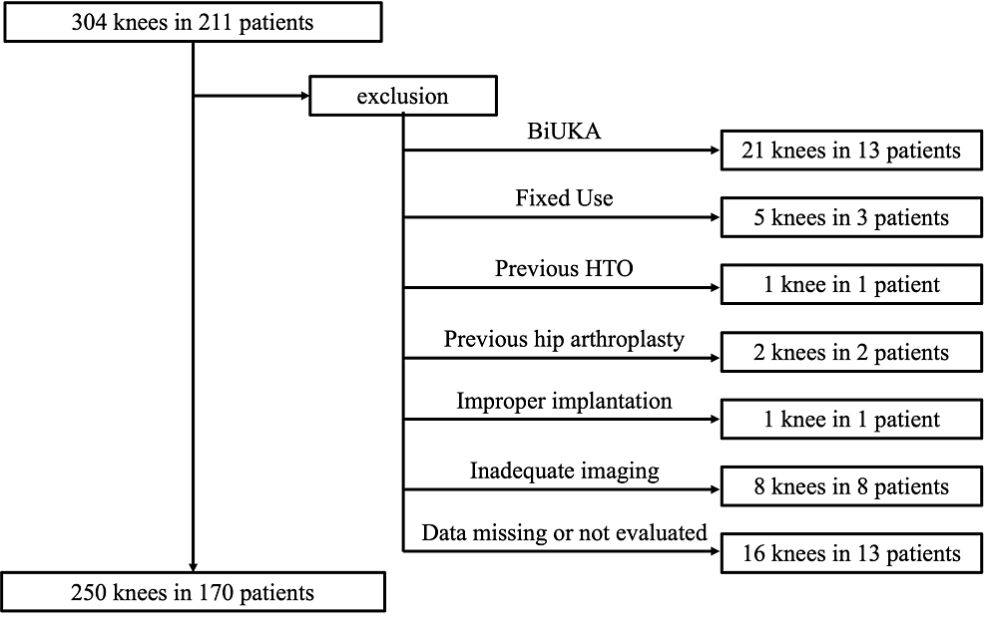
Patient recruitment flowchart. BiUKA: bi-unicompartmental knee arthroplasty, HTO: high tibial osteotomy.

Here, 248 knees from 166 patients were included (men: 51; women: 115; age range: 49 to 89 years; mean age: 74.3 years) (Figure 2).

All measurements are shown in Table 1.

**Table 1:** Radiographic measurements. n = 250 knees (in 170 patients); aHKA was compared to postoperative mHKA. ^a^Difference in pre- and postoperative LDFAs vs. difference in pre- and postoperative MPTAs: P < 0.059, Hedges’ g: 0.140 ^b^Difference in pre-and postoperative mHKA vs. difference between preoperative aHKA and postoperative mHKA: P < 0.001, Hedges’ g: 1.500. aHKA: arithmetic hip-knee-angle angle, mHKA: mechanical hip-knee-ankle angle, LDFA: lateral distal femoral angle, MPTA: medial proximal tibial angle.

	Preoperative	Postoperative	P-value	Difference	Hedges' g
LDFA	1.13° ± 2.35°	1.83° ± 2.34°	<0.001	0.70° ± 2.37°^a^	0.298
MPTA	−5.21° ± 2.31°	−6.28° ± 2.76°	<0.001	−1.07° ± 2.54°^a^	0.418
mHKA	−9.04° ± 4.21°	−4.46° ± 3.29°	<0.001	4.58° ± 3.60°^b^	1.208
aHKA	−4.08° ± 3.17°		<0.001	−0.38° ± 2.96°^b^	0.117
JLO	−6.33° ± 3.42°	−8.97° ± 3.92°	<0.001	−1.77° ± 3.85°	0.481

**Figure 3:**
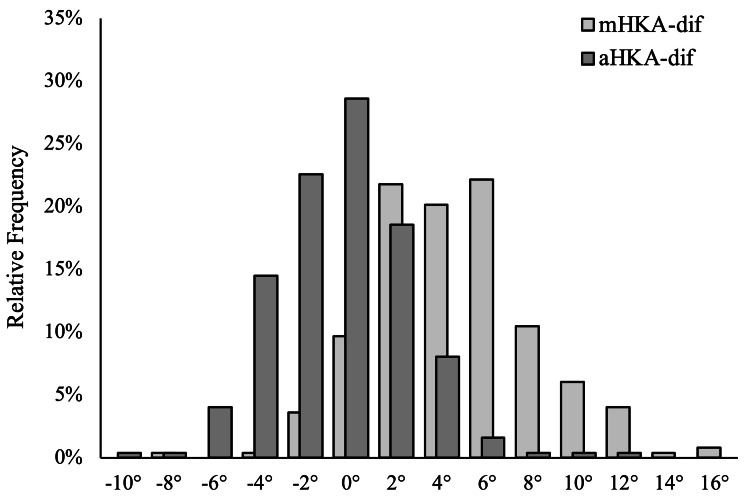
The difference between preoperative mHKA and postoperative mHKA (mHKA-dif) and the difference between preoperative aHKA and postoperative mHKA (aHKA-dif). The aHKA-dif distributes around 0°, whereas the mHKA-dif distributes positively, showing corrections were made toward values postoperatively.

The preoperative mHKA, preoperative aHKA, and postoperative mHKA were −9.04° ± 4.21°, −4.08 ± 3.17°, and −4.46° ± 3.29°, respectively (Figure 1). The aHKA-dif was −0.38° ± 2.96°, whereas the mHKA-dif was 4.58 ± 3.60° (Figure 3). The difference between the aHKA-dif and the mHKA-dif was statistically significant (P < 0.001). The postoperative mHKA was significantly different from the preoperative mHKA (P < 0.001) with a large effect size (Hedges’ g, −1.21; 95% confidence interval (CI): −1.37 to −1.052). Similarly, the preoperative aHKA was significantly different from the postoperative mHKA (P = 0.04), albeit with a small effect size (Hedges’ g, 0.117; 95% CI: 0.003 to 0.232) (Table 1).

Both the pre-aHKA (r = 0.58; 95% CI: 0.492 to 0.657; P < 0.001) and the pre-mHKA (r = 0.564; 95% CI: 0.473 to 0.643; P < 0.001) correlated with the post-mHKA.

**Figure 4:**
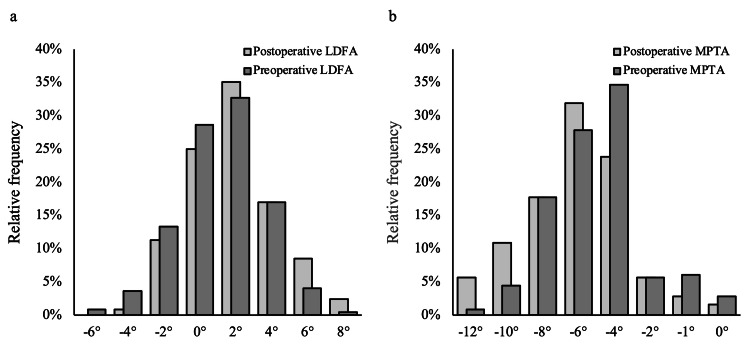
Distributions of pre and postoperative LDFA (a) and MPTA (b). Both LDFA and MPTA tended to be valgus postoperatively. LDFA: lateral distal femoral angle, MPTA: medial proximal tibial angle.

The LDFA tended to be more valgus postoperatively (preoperative LDFA: 1.83° ± 2.34°; postoperative LDFA: 1.13° ± 2.35°; P < 0.001) (Figure 4a); similarly, the MPTA tended to be more valgus postoperatively (preoperative MPTA: −6.28° ± 2.76°; postoperative MPTA: −5.21° ± 2.31°; P < 0.001) (Figure 4b).

**Figure 5:**
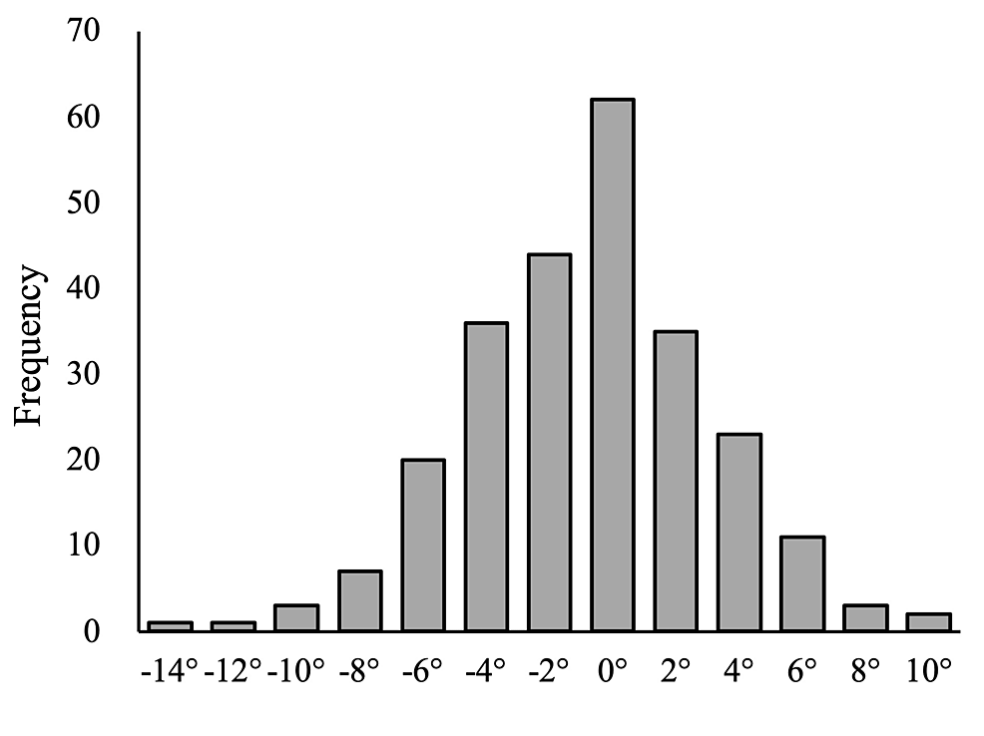
The JLO change (postoperative JLO - preoperative JLO) in Oxford UKA. The JLO tended to incline medially (apex-distal) postoperatively. JLO: joint line obliquity.

The change in the MPTA (1.07° ± 2.54°) tended to be larger than that in the LDFA (0.70° ± 2.53°), showing statistical insignificance (P = 0.059). The preoperative and postoperative JLO were −6.33° ± 3.42° and −8.97° ± 3.92°, respectively, with a significant difference (P < 0.001; Hedges’ g: 0.481) (Figure 5).

## Discussion

The most important finding of this study was that the preoperative aHKA was similar to the postoperative mHKA. Therefore, preoperative aHKA is a good predictor of mHKA after mobile-bearing Oxford UKA. The alignment strategy of Oxford UKA is to restore the leg alignment to the patient’s pre-disease (or native, constitutional) alignment rather than reconstruct the leg alignment to a target alignment (neutral or slightly varus) [8]. In a previous study on patients having unilateral arthritis with a healthy opposite knee, the aHKA was similar to the healthy mHKA, which indicates pre-disease leg alignment [22]. Our study showed that the postoperative mHKA was equivalent to the preoperative aHKA [17]. Hence, the leg alignment after Oxford UKA is considered similar to the patient’s CA. This is the first study to show that postoperative leg alignment can be predicted by the preoperative measurements in Oxford UKA.

Although there were no significant differences between the preoperative aHKA and the postoperative mHKA, and the mean difference between them was −0.4° ± 3.0°, the predictive ability was imperfect, and approximately 30% of the knees showed a difference of >3°. Mullaji et al. reported that 83% of the knees after Oxford UKA were within ±3° of the contralateral healthy knees [22]. Griffiths-Jones et al. reported that the difference between the aHKA of arthritic knees and the mHKA of healthy knees was −0.4° ± 0.4° [17]. Although the mean values are equivalent, the variation in our study was larger than that in previous studies. This variation might be caused by several factors. The gap, especially in extension, plays a significant role in determining the amount of milling, the size of the bearing, and eventual postoperative leg alignment [23]. The evaluation was performed manually using feeler gauges, which can be a source of variation. Similarly, evaluating the extension gap at 20° flexion can also influence the alignment in extension because approximately 40% of the knees have a looser full-extension gap than the 20° flexion gap [24]. Moreover, the aHKA can be affected by bone defects. Nevertheless, the aHKA is a good predictor of the postoperative mHKA compared with the preoperative mHKA. Although there were similar relationships between the preoperative aHKA and the postoperative mHKA and the preoperative mHKA and the postoperative mHKA, the latter difference is larger than the former difference; hence, it cannot be used for postoperative mHKA prediction.

Interestingly, postoperatively, the LDFA tended to be valgus, and the MPTA tended to be varus. The differences were approximately 1°, and they compensated for each other. Eventually, the JLO inclined more medially by approximately 1.7°. Theoretically, the joint line is not expected to change; however, the results indicate that the joint line tended to be more medially inclined postoperatively. There has been no previous study on joint line alteration after Oxford UKA. Only one report by Nishida et al. showed that the medially inclined joint line orientation (angle between the joint line and the floor) was associated with a declined postoperative clinical outcome [25], indicating that the joint line alteration can affect the clinical outcome. Further investigation is required to clarify the relationship between changes in LDFA and MPTA and the clinical outcome.

Limitations

This study has some limitations. First, this study was two-dimensional. Although the protocol was strictly established, the imaging technique can affect the results. We strictly excluded any inadequate images due to the rotation of the legs; however, other deformities, such as flexion contracture and image distortion, could also have affected the measurement. The 3D-CT analysis would be ideal; however, its radiation exposure and cost are problematic. Second, there was no evaluation of the clinical outcome. Postoperative alignment, especially in the joint line, can affect the postoperative clinical outcome. Third, this is a retrospective study; a prospective study might be required to confirm the reliability of the prediction. Finally, and most importantly, we did not evaluate the clinical significance. Further investigation is required to evaluate the impact of the postoperative alignment and the difference between the postoperative alignment and the estimated one on the clinical outcome and implant survivorship.

Despite these limitations, the preoperative aHKA can, to some extent, be a predictor of postoperative lower limb alignment. Future studies will aim to clarify the relationship between the postoperative alignment change and the clinical outcome and implant survival.

## Conclusions

The preoperative aHKA was found to be similar to the postoperative mHKA. Therefore, it can be regarded as a predictor of postoperative leg alignment after Oxford UKA. Meanwhile, the joint line inclined medially postoperatively. Further investigation is required to evaluate the effect of joint line alteration.
